# Sex discriminations made on the basis of ambiguous visual cues can be affected by the presence of an olfactory cue

**DOI:** 10.1186/2050-7283-1-10

**Published:** 2013-06-19

**Authors:** Graeme Hacker, Anna Brooks, Rick van der Zwan

**Affiliations:** Laboratory of Cognitive Neuroscience and Behaviour Southern Cross University, Coffs Harbour Campus, Hogbin Drive, Coffs Harbour, NSW 2450 Australia

**Keywords:** Sex perception, Human pheromones, Multi sensory perception

## Abstract

**Background:**

Almost every interpersonal interaction is mediated by the sex of the individuals involved. Visual, auditory, and olfactory cues provide individuals with the opportunity to discriminate the sex of others from a distance and so prepare sex-appropriate behaviours for any impending interaction. The usefulness of that important social skill is mediated by the reliability of the sensory information. Sometimes cues in one domain will be ambiguous, and the perceptual processes mediating sex perceptions will need to integrate information from across the senses for better reliability. With that in mind, the experiment reported here was designed to explore the effect of olfactory-visual interactions on sex perceptions.

**Methods:**

Observers were presented visually with point-light walkers that were sexually ambiguous (not unequivocally female or male). They were asked to judge, using a two-alternative forced choice paradigm, the sex of each walker. Tested on two occasions, observers unknowingly made sex judgements in the presence or absence of pads soaked in male sweat.

**Results:**

The presence of male sweat was associated with higher proportions of ‘male’ judgements of both ambiguous female and ambiguous male walkers (F_1,19_ = 24.11, *p* < 0.01).

**Conclusion:**

These findings suggest that olfactory cues can modulate visual sex discriminations made on the basis of biological motion cues. Importantly, they seem to do so even when the olfactory cue is not consciously perceived, suggesting these effects are mediated by perceptual rather than cognitive processes.

These findings suggest that there exist cortical processes mediating sex perceptions that are capable of integrating visual and olfactory information. What is important is that this sensory integration takes place without conscious knowledge and that appropriate behaviour modifications may occur automatically.

## Background

Sex perception, the ability to discriminate accurately the sex of an observed other, is a central prerequisite for all interpersonal interactions. Of particular interest here are the cues that modulate sex perceptions from multisensory input. Work using various types of stimuli suggests the existence of “sex tuned” neurons (Jordan et al. 2006; 2005; Troje et al. 2006 at least some of which are multi-sensory [see also Eagleman 2001; Shimojo & Shams 2001; Kovacs et al. 2004; van der Zwan et al. 2009)]. Of those studies two are particularly interesting in the present context. Using combinations of olfactory and visual cues (Kovacs et al. 2004) showed that both male and female olfactory cues make sexually ambiguous faces appear more often to be, respectively, male or female. Similarly, (van der Zwan et al. 2009) combined unambiguous auditory sex-cues with ambiguous visual sex-cues to show perceptual integration: The sound of female footsteps made sexually ambiguous point-light walkers (Johansson 1973) appear more often to be female (van der Zwan et al. 2009).

Two similarities between those two studies immediately are apparent. Both paired visually ambiguous sex cues with a cue from a second modality that was both sexually unambiguous and consciously perceived. For example, (Kovacs et al. 2004) presented volatile sex hormone-like steroids, androstadienone and estra-tetraen-ol, mixed into a scented paste, caused observers to resolve sexually ambiguous faces into specific sex categories. While the classification of these olfactory stimuli as human pheromones is still only supposition, androstadienone did shift observer’s perceptions of sexually ambiguous faces such that they more often were judged to be male. Similarly, estra-tetraen-ol caused observers more often to perceive sexually ambiguous faces as female. Those observations were interpreted as showing that observers could use an unambiguous olfactory sex cue to resolve ambiguity in a visual cue to give rise to unambiguous sex perceptions (Kovacs et al. 2004).

In much the same way, van der Zwan et al. (2009) used auditory representations to shift observer’s vision-based perceptions of sexually ambiguous point-light walkers (Johansson 1973). van der Zwan et al. (2009) developed an auditory walking sequence (a series of foot-falls) that observers reliably rated as sounding female. They went on to show, using an aftereffects paradigm, that when that auditory walking sequence was paired with a sexually ambiguous visual walker, observers would subsequently report that walker to be male. Those data too were interpreted as evidence that observers confronted with visually ambiguous sex information could use an unambiguous and consciously perceived cue from another modality to resolve perceived sex.

While there is precedent for auditory cues affecting visual perceptions (2009) the capacity for neural processes to use *olfactory* cues to resolve visual ambiguities is less well understood. To that end, a discussion of the level at which olfactory/visual interactive processing might occur can provide some insights. For example, the capacity for combined stimuli to induce aftereffects has been taken as evidence of true perceptual integration (Ernst & Bülthoff 2004;Ernst 2006). Similarly, the capacity for sub-threshold stimuli mutually to influence resulting perceptions can be interpreted as evidence that the processes by which that integration occurs are perceptual rather than, say, cognitive.

With that in mind, the experiment reported here was designed to further explore the nature of olfactory-visual interactions. A number of studies have shown that olfactory cues, even when they are not consciously perceived, can affect physiological processing and behaviours (2007; Lundstrom et al. 2003; Lundstrom & Olsson 2005). To our knowledge, what has not previously been shown is whether sub-threshold olfactory cues can be used to mediate sex perceptions and specifically, visual sex perceptions in the same way. Thus, the aim of this experiment was to determine whether a sub-threshold olfactory sex cue could affect perceptions in the same way as such cues have been shown to affect mood and arousal. Specifically, this experiment tested the hypothesis that observers would more often judge visually ambiguous walkers to be male when performing the task in the presence of male sweat, compared to when the olfactory cue was absent. Further, we predicted they would do so even when not able to report the presence of the sweat odorant.

## Methods

### Participants

20 participants (12 females & 8 males) aged between 18 and 50 (M = 27, SD = 8) were recruited from Southern Cross University (Coffs Harbour) campus. Participants were naive to the aims of the experiment. Participants had normal to corrected vision, none were anosmic, and none were ill at the time of testing. Informed written consent was obtained from all participants prior to experimentation. This study was conducted in compliance with the Helsinki Declaration and was approved by the Human Research Ethics Committee of Southern Cross University ECN-10-138.

### Materials

Participants were seated in front of a Dell Trinitron flat-screen monitor at a viewing distance of 57 cm in an unlit, sound-attenuated testing cubicle. Data was collected on a Pentium 4 processor. The display resolution of the monitor was set to 1,024 × 768 pixels, it was calibrated for luminance, and had a refresh rate of 100 Hz at 32 bit colour resolution. Participant responses were recorded using a Microsoft Wireless Multimedia Keyboard 1.0A. Participants signalled their responses (“male” or “female”) using the “m” and “z” keys, counter-balanced across participants.

#### Visual stimuli

PointLightLab (v4.0.13) custom-written software was used to generate the visual stimuli. The point-light walker stimuli used here were derived from examples of walkers taken from the gender-continuum developed by Troje (2002; Troje 2008). Detailed methods describing how the walkers of which that continuum is composed were created have been provided elsewhere (Troje 2008). In summary: Increments along the gender continuum were obtained first by integrating the gaits of 50 female and 50 male walkers. Then, using linear classifiers derived from the female and male subsets respectively, standard deviations from the mathematically average walker (represented as 0) were calculated to create walkers that were more female (increments below 0 on the continuum) and more male (increments above 0). Thus, increment 0 is the statistically neutral walker at the centre of the continuum and the statistical “sex” of each increment away from 0, both in the female and male directions along the continuum, was calculated.

Using PointlightLab to generate animated point-light walkers based on Troje’s models this experiment used as test stimuli the most perceptually ambiguous female and male exemplars from that continuum. Pilot studies and other work from the laboratory had shown that on a 13 increment continuum, numbered from −6 (extreme female walker) to +6 (extreme male walker), the walkers at the −1 and 0 positions are most often judged as being the most sexually ambiguous. As noted above, the 0 walker is the statistically neutral walker. Observers typically report the 0 walker as looking slightly male (congruent with the so-called male-bias observed for point-light walkers: (Troje 2008); and for faces: (Davidenko 2007)) and so it was included here as a test stimulus because it represented to most ambiguous walker with perceptually male characteristics. This stimulus will be, here, described as the “*ambiguous male*” walker. The −1 walker on the continuum, the first walker with any female characteristics, is typically the increment closest to the perceptually neutral point on Troje’s continuum (van der Zwan et al. 2009; Troje 2008). In contrast to the 0 walker, this walker typically is reported by observers to look slightly female. It was included as a test stimulus here because it is the most ambiguous walker to carry slightly female characteristics. This stimulus will be, here, described as the “*ambiguous female*” walker.

Both the ambiguous male and the ambiguous female walkers were constructed using 15 white points defining the major joints of a human actor (wrists, elbows, shoulders, centre sternum, hips, middle pelvis, knees, ankles) plus three additional reference points (centre sternum, centre pelvic, and head). Each walker was presented on a black background. Walkers were orientated on the frontal plane so as to face away/towards the observer (direction-of-facing is, in objective terms at least perfectly ambiguous, but see also (Schouten et al. 2010)). Each walker was shown in motion, walking as if on a treadmill (they neither loomed nor receded). Each dot of which the models were composed subtended a visual angle of 0.3° and the PLWs stood 20.5° visual angle high and 6.5° wide. To reduce the ability of participants to monitor just a single spatial location PLWs were on each presentation positioned randomly within a pre-assigned region of uncertainty (a square 10° × 10° visual angle).

Trials containing target stimuli had interspersed between them presentations of distractor walkers, each sexually unambiguous. Distractor walkers were constructed using exactly the same methods as the target stimuli but were chosen from more extreme locations on Troje’s (2002; Troje 2008) continuum to ensure they were not sexually ambiguous. Female and male distractors occurred with equivalent frequency. The target/distractor ratio was 0.22.

#### Olfactory stimuli

Lundstrom and Olsson (2005) demonstrated that congruency between the environment and a pheromone-like chemosensory cue is important for behavioural effects to be conveyed via olfaction. Because there was only one male experimenter running the trials it was decided that using a female chemosensory cue (sweat) could be confounded by the overall conditions. For this reason congruency between the environment (male experimenter) and the olfactory cue used was maintained and only male sweat was used.

The criteria and donor requirements for sweat collection were based on methods used by Zhou and Chen (2009). Fresh sweat samples were collected each day of testing in order to minimise bacterial growth and the consequent odour. Each time, samples were taken from two of three possible donors using cotton pads taped under the arms and to the abdomens of the male donors while they exercised (running or cycling for 40 – 60 mins). For 24 h prior to collection, donors avoided consuming foods known to add odorants to sweat (eg garlic, chilli, asparagus). For that same period they abstained from having sex, and from applying deodorants or strong scented soaps. All male sweat donors were healthy adults with no medical conditions and were taking no medications. Donors were also required to be available to participate for the duration of the study.

Fifteen minutes before testing commenced the sweat-soaked cotton pads were collected and placed into an opaque plastic bowl that was then placed in the testing cubicle behind the computer monitor, 80-100 cm from the participant. The container was placed out of the direct line-of-sight of the participant to avoid potentially priming participants as to the purpose of the experiment. Sweat pads from more than one donor always were combined to avoid the possibility of interactions between an individual donor and participants.

### Procedure

Participants completed the sex-discrimination task on two separate occasions: Once for the non-olfactory condition (no sweat cue) and once with the olfactory (sweat) cue present. On all occasions participants were kept naïve as to the nature of the experiment, specifically that it included an olfactory manipulation. The order of the two conditions was counter-balanced across participants.

Upon arrival at the laboratory participants were asked to sit in front of a computer screen in an unlit cubicle. Lundstrom and Olsson (2005) previously have reported that some effects are contingent on social context and that effects mediated by male olfactory cues are most effective when the experimenter is male. For that reason a male experimenter was used on all occasions. Having given instructions and answered any questions the experimenter immediately left the testing cubicle and closed the door. The experimenter then monitored proceedings using a slave monitor in an adjacent room. Presentation protocol comprised a 3 second blank grey pre-stimulus screen followed by a 1000 msec presentation of a walker (one complete 2-step walking cycle). Participants recorded their response in a post-stimulus 5 second interval. Participants completed on each occasion two blocks of trials, each composed of 11 different walkers (2 target walkers, 9 distractors) each repeated 5 times. Participants were given a short break between blocks of trials.

Having completed their first session participants arranged to return, a week later, for a second testing session where the procedures were repeated. The olfactory cue was present at only one session but on all occasions identical plastic containers to the one in which pads were stored was in place behind the computer monitor – a measure implemented (in spite of participants’ naivety to the olfactory manipulation) to provide the most stringent possible control across conditions.

After the second testing session participants were debriefed without being told of the aim of the experiment or of the presence or otherwise of the olfactory cue. All were questioned, during debriefing, about their visual, auditory, temperature, and olfactory experiences as an observer. No participant reported any awareness of unusual odours or odorants in the testing room.

## Results

**Figure 1:**
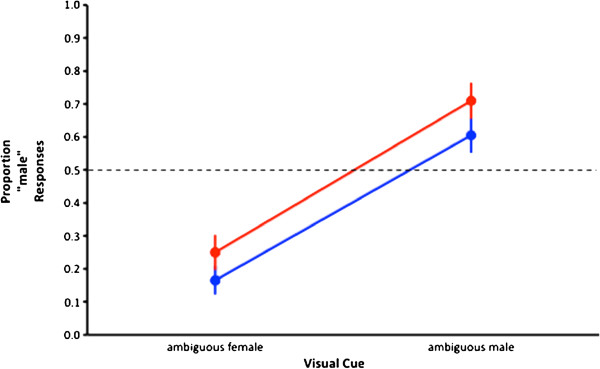
**Olfactory influence on perception.** The effects of an olfactory cue (male sweat) on visual sex discriminations of sexually ambiguous point-light walkers. The blue function describes average performances on the ambiguous female and male walkers in the absence of the manipulated olfactory cue. The red function describes the average performances when the cue was present. The broken black line indicates chance performance. Clearly female walkers were judged to look female more often than they were judged as being male. Male walkers, conversely, were judged as being male more often than female. The presence of male sweat increased the proportions of times both female and male walkers were judged to be male even when participants were not aware of the presence of the cue. Bars indicate ± 1 standard error.

The mean performance for each participant on each condition was calculated as the average of the 10 separate presentations of each target stimulus (ambiguous female, ambiguous male, for both olfactory and non-olfactory conditions). We found no differences between female and male observers, and no order effects so data were collapsed across participants for each conditions and means and standard errors calculated (Figure 1). As those data show, the mean proportion of times the ambiguous female walker, in the absence of the olfactory cue, was judged to be “male” was 0.17 ± 0.04. The mean proportion of times the ambiguous male was judged, in the absence of olfactory cues, to be male was 0.61 ± 0.05. Both proportions increased in the presence of the olfactory cue: In the presence of male sweat the mean proportion of times the ambiguous female walker was judged to be male was 0.25 ± 0.05, an increase of 8%. Similarly, the mean proportion of times the ambiguous male was judged to be male increased, in the presence of the olfactory cue, to 0.71 ± 0.05, an increase of 10%.

An analysis of variance for repeated measures on two factors revealed that there were significantly more “male” responses made for the ambiguous male walkers than for the ambiguous female walkers (F_1,19_ = 24.11, *p* < 0.01). Importantly, the presence of the male sweat olfactory cue significantly increased the proportions of “male” responses for both ambiguous female and ambiguous male walkers (F_1,19_ = 3.10, *p* < 0.05). There was no significant interaction between the two variables (F_1,19_ = 0.11, *p* > 0.05). That is, and as predicted, these data provide evidence that the presence of male sweat increased the proportions of times observers judged ambiguous walkers to be male. This suggests the odorant influenced participants’ resolution of visual ambiguities when determining the sex of the walkers they were observing.

No participant indicated any awareness of the olfactory cue: Not one individual, during debriefing, noted the presence of any smells at all. As such the olfactory cue was at the very least non-salient (and perhaps, although it cannot categorically be stated, sub-threshold). That observation represents an important difference between this experiment and earlier work. It suggests a remarkable ability to exploit even those sensory cues of which observers are not consciously aware to resolve ambiguities inherent in other sensory domains.

## Discussion

The general aim of this experiment was to determine whether an olfactory sex cue that is not consciously perceived would affect perceptions in the same way as supra threshold olfactory cues have been shown to affect mood and arousal. Specifically, the experiment reported here tested the hypothesis that olfactory cues contained in male sweat would be used, by observers, to mediate sex perceptions of sexually ambiguous walkers. Sexually ambiguous point-light walkers were presented to observers who had to discriminate each as either female or male and observers made their judgements both in the presence and in the absence of male sweat. The data show that even though participants were unaware of changes in the olfactory landscape, the presence of male sweat systematically influenced (increased) the proportions of times observers judged sexually ambiguous walkers to be male.

A number of important implications arise from these data. First, they extend existing reports that in perceptually ambiguous environments olfactory cues can mediate other-modality perceptions (Kovacs et al. 2004; Zhou & Chen 2009; Mujica-Parodi et al. 2009) into the realm of sex perception. Based on the importance of that social skill - one that underpins almost every imaginable inter-personal interaction (Stangor et al. 1992) – the observation that multimodal processing is involved is perhaps unsurprising: Even the most reliable cue sometimes will contain ambiguity (as demonstrated with the visual stimuli used here) and the ability to resolve ambiguities in one sense with information from another maximises the advantage conveyed by the mechanisms using those cues. Accordingly, multimodal integration has so far been evidenced in relation to a number of socially relevant tasks (see for example (Ernst 2006; Bresciani et al. 2008)). Yet there has not previously been any demonstration of the capacity for human observers to integrate olfactory information into perceptions of sex based on visual biological motion cues. The present data provide preliminary evidence consistent with the suggestion that perceptions of visually ambiguous gaits can be integrated with olfactory cues (in this case those contained in male sweat) to change the quality of visually perceived sex.

The demonstration that even olfactory cues that are not consciously perceived can influence visual perceptions adds an additional element. A number of behaviours are affected by sub-threshold concentrations of some active olfactory cues (Lundstrom et al. 2003; Bensafi et al. 2003; Wyart et al. 2007), but here for the first time is evidence that such effects occur in relation to sex processing from biological motion cues. Such a finding has its own important implications. Specifically, it is in keeping with the proposition that the integration of multimodal sex cues takes place at a perceptual rather than cognitive level; that the mechanisms via which integration is achieved operate automatically. The suggestion that such processes are perceptual, and so might be thought of as requiring no conscious effort by the observer, is novel with respect to olfactory/visual sex cues, but has precedent in literature relating to other multimodal cue combinations (see for e.g. (van der Zwan et al. 2009)).

That there are in place neural processes that subserve the perceptual integration of olfactory and visual cues gives rise to a number of further possibilities. It may be, for example, that that the sex perception mechanisms mediating the effects reported here reflect cue-invariant mechanisms integrating olfactory and visual cues to sex. As noted above, however, those processes and their neural loci as yet remain unclear. Bayesian Decision Theory has been shown to be useful for modelling the interactions between senses and experience that give rise to perceptions and there is some evidence that Bayesian decision theory can equally well be applied to the integration of olfactory with visual cues (Shankar et al. 2010). The real advantage of that approach is that, using Bayesian techniques, it is possible to separate out behavioural effects attributable to probability summation from effects attributable to real perceptual integration. That is, the presence of cues simultaneously in two sensory domains (here a cue in the olfactory domain presented simultaneously with one presented in the visual domain) increases the likelihood through a process of summation: Each stimulus gives rise to a certain probability of a response and the two cues together mean those two probabilities add. However, real sensory integration, or perceptual binding such that the cues in each domain are perceived as coming from a single source, give facilitatory effects above those of simple cue summation. To our knowledge though there has to date been no attempt to model, using Bayesian decision theory, the mechanisms underpinning sex perceptions involving olfactory and other cues to see if binding does occur.

In that context, future investigations should clarify more fully these findings by testing for equivalent effects with female olfactory cues under the correct contextual conditions, and by systematically titrating the objective concentration levels of each type of olfactory signal and observing resulting multisensory perceptual response functions. Those experiments are not trivial, needing to balance the fertility states of the donors and experimenter. Nonetheless, such manipulations will allow for a clearer picture to emerge of the perceptual and neural substrates underpinning olfactory/visual cue integration during the task of discriminating sex. They will clarify also the generalisability of the effects reported here.

## Conclusion

To conclude, these results are preliminary evidence for the existence of cortical processes mediating sex perceptions capable of integrating visual and olfactory information. It is noteworthy that this sensory integration seems to takes place without conscious knowledge and may indeed lead to automatic behavioural modifications that best suit the social surroundings.
